# Endobronchial lipoma

**DOI:** 10.4322/acr.2021.377

**Published:** 2022-05-02

**Authors:** Lakshitha Anbazhakan, Asad Ullah, Rohit Munagala, Rabih Bechara, Islam Elhelf, Nikhil Patel, Nagla Abdel Karim

**Affiliations:** 1 Augusta University, Medical College of Georgia, Augusta, Georgia, USA

**Keywords:** Lipoma, Bronchoscopy, Airway Obstruction

## Abstract

Endobronchial lipomas are rare benign lung tumors that can cause bronchial obstruction and parenchymal damage. While an uncommon etiology, they are often misdiagnosed due to a clinical presentation similar to obstructive pulmonary pathologies such as COPD and asthma. Upon review of English-language literature, under 50 cases of endobronchial lipomas were documented in the prior 10 years (2011-2021). There are no clear guidelines regarding the management of this particular entity, but typically interventional debulking is the treatment of choice. Here we present another unique case of endobronchial lipoma along with our diagnostic and therapeutic methodology. The patient underwent bronchoscopic debulking via a cryotherapy probe. Based on the histopathologic analysis, a diagnosis of endobronchial lipoma was made. Endobronchial lipomas must remain in any clinician’s differential when a patient presents with dyspnea. We report the unique location of this lipoma based on our literature review and the importance of investigating endobronchial lesions due to a possible diagnosis of endobronchial lipoma.

## INTRODUCTION

Endobronchial lipomas are rare benign lung tumors that can cause bronchial obstruction and parenchymal damage. They can mimic chronic obstructive pulmonary disease (COPD) or asthma.^1^ Diagnosis is made by imaging studies, mainly computed tomography scan (CT scan), and then confirmed by a biopsy. Early diagnosis is essential to prevent possible bronchial obstruction or further complications. Bronchoscopic resection has now become the preferred mode of treatment versus surgical intervention.^1^

## METHODS

A search on PubMed was conducted using the keywords “endobronchial lipoma.” Only articles between 2011-2021 were considered. At the time of search under these parameters, there were 48 results. Of the 48 results, 35 articles, including case reports or any mention of a patient with endobronchial lipoma, were used. Foreign articles were used only if the full article was written in English. Articles reporting myxomas or hamartomas without specifying endobronchial lipoma were excluded. One article was used that included 4 case reports, 2 of which were reported to be endobronchial lipoma.^2^ Any meta-analysis or clinicopathological reviews were also excluded from Table 1.

**Table 1:** Review of Other Cases of Endobronchial Lipoma

ref	Age (Y) / Gender	Location	Treatment
1	71/M	Left upper lobe bronchus	Flexible bronchoscope with electrocautery snare
3	63/M	Left main bronchus	Snare electrocautery, tumor debulking, and argon plasma coagulation
4	63/F	Left lower lobe bronchus	Not mentioned
5	56/M	Left lobe main bronchus	Bronchoscopic excision
6	73/M	Left main bronchus	Flexible bronchoscopy with snare electrocautery, bronchoalveolar lavage
7	64/M	Entrance of Right upper lobe	Thoracotomy with upper right lobectomy and lymphadenectomy
8	63/M	Distal lateral wall of the bronchus intermedius and superior segment of the right lower lobe	Rigid bronchoscopy with forceps, scissors, and snare electrocautery
9	61/M	Left lower lobe bronchus	Electrocautery snare followed by argon plasma coagulation at the base of the tumor
10	82/M	Right main bronchus	Limited bronchoscopic resection, further mass resection, mediastinal dissection, and bronchoplasty
11	63/M	Left lower lobe bronchus	Left lower lobectomy
12	74/M	Right main bronchus	Bronchoscopic mass resection
	44/F	Right inferior lobe basal trunk bronchus	Right inferior lobe resection
13	43/F	Right lower bronchus	Not mentioned
14	63/M	Left main bronchus	Laser resection
15	83/M	Right main bronchus	Flexible bronchoscope with electrocautery snare
16	83/M	Right lower lobe bronchus	Electrocautery snare
	35/M	Right main bronchus	Not mentioned
17	67/M	Right lower lobe - superior segmental bronchi	Cryorecanalization
18	60/M	Right lower lobe - upper segment	Right lower lobe upper-segmentectomy with hilar and mediastinal lymphadenectomy
19	65/M	Right upper bronchus	Right thoracotomy, right upper lobe resection
20	48/F	Right main bronchus	Initially flexible bronchoscope excision, then extraction with rigid bronchoscopic forceps
21	52/M	Left main stem bronchi and left upper lobe bronchus	Electrocautery snare and argon plasma coagulation
22	72/M	Subsegment of the left posterior basal segment	Bronchoscopic resection was proposed during initial endoscopic procedure, but it was denied by the patient
23	66/F	Lateral segmental bronchus of the right lower lobe	Uniportal thoracoscopic right basal segmentectomy
24	63/M	Basal segmental bronchi of the left lower lobe	Endobronchial resection by laser and cryotherapy
25	60/M	Left upper lobe bronchus	Limited surgical resection
26	54/M	Right main bronchus	Pneumonectomy
27	52/M	Left main bronchus and the superior segment of the left lower lobe	Electrosurgical snare, cryotherapy, and electrocautery
28	68/?	Right-lower-lobe segment	Right-lower lobectomy
29	62/M	Left upper lobe bronchus	Flexible bronchoscopic electrosurgical snare, additional coagulation using snare tip
2	39/M	Posterior segment of the left lower lobe	Flexible bronchoscope, a polypectomy snare, and electrocautery
	78/F	Right lower lobe bronchus	Flexible bronchoscope, a polypectomy snare, and electrocautery
30	69/M	Right intermediary bronchus	Resection via Percutaneous Gastrostomy Snare Device
31	69/M	Anterior segmental bronchus of the right upper lobe	Resection via flexible bronchoscopy and cryotherapy probe
32	70/F	Bifurcation of the left-upper and lower lobe bronchi	Rigid endoscopy

Ref= reference

## RESULTS

We reviewed the literature between 2011-2021. Of the 32 articles that were encountered, there were a total of 35 reports of patients with endobronchial lipoma (Table 1).

Of the reported cases, 17 cases were of the left lung bronchi, and 20 were of the right lung bronchi (Table 2). One of the cases detailed an endobronchial lipoma at the bifurcation of the left upper and lower lobe bronchi. The majority of cases involved patients that were ≥ 60 years old (26 cases) and male (27 cases). One article did not identify the patient’s gender. Most were treated with bronchoscopic resection (electrocautery, laser). There were 3 articles that did not specify their method of treatment.

**Table 2:** Location of Endobronchial Lipoma

Location	# Of Cases	Location	# Of Cases
Right main bronchus	6	Left main bronchus	6
Right upper bronchi	3	Left upper bronchi	4
Right intermediary bronchus	2	Left lower bronchi	7
Right middle bronchi	0		
Right lower bronchi	9		
Total	20	Total	17

# = number.

## CASE REPORT

We present a 70-year-old male patient who had progressive dyspnea on exertion, chest pain, and lightheadedness 2 months after bicuspid aortic valve replacement surgery. He had routine imaging studies pre-operatively. His past medical history was significant for hypertension, atrial fibrillation, obstructive sleep apnea, hyperlipidemia, chronic diverticulitis, and sigmoid abscess post colectomy.

**Figure 1:**
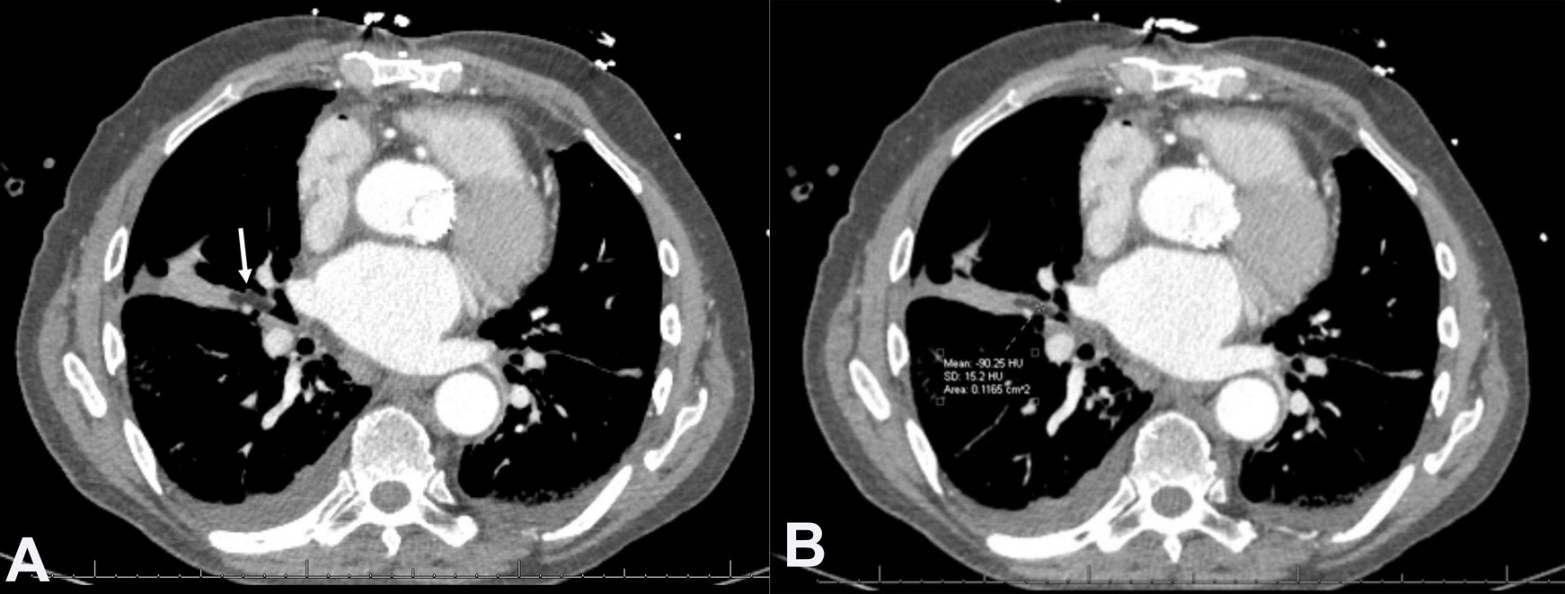
CT scan of the chest with IV contrast (A) Axial image shows a proximal right middle lobe bronchus hypodense lesion (arrow). (B) The lesion displays negative Hounsfield units (-90 HU) consistent with fat component.

CT scan of the chest revealed a hypodense lesion in the right middle lobe bronchus with negative Hounsfield values consistent with the macroscopic fat component (Figure 1A). Subsequently, endobronchial ultrasound (EBUS) was indicated, where a right middle lobe mass was found with 99% obstruction (Figure 1B).

**Figure 2:**
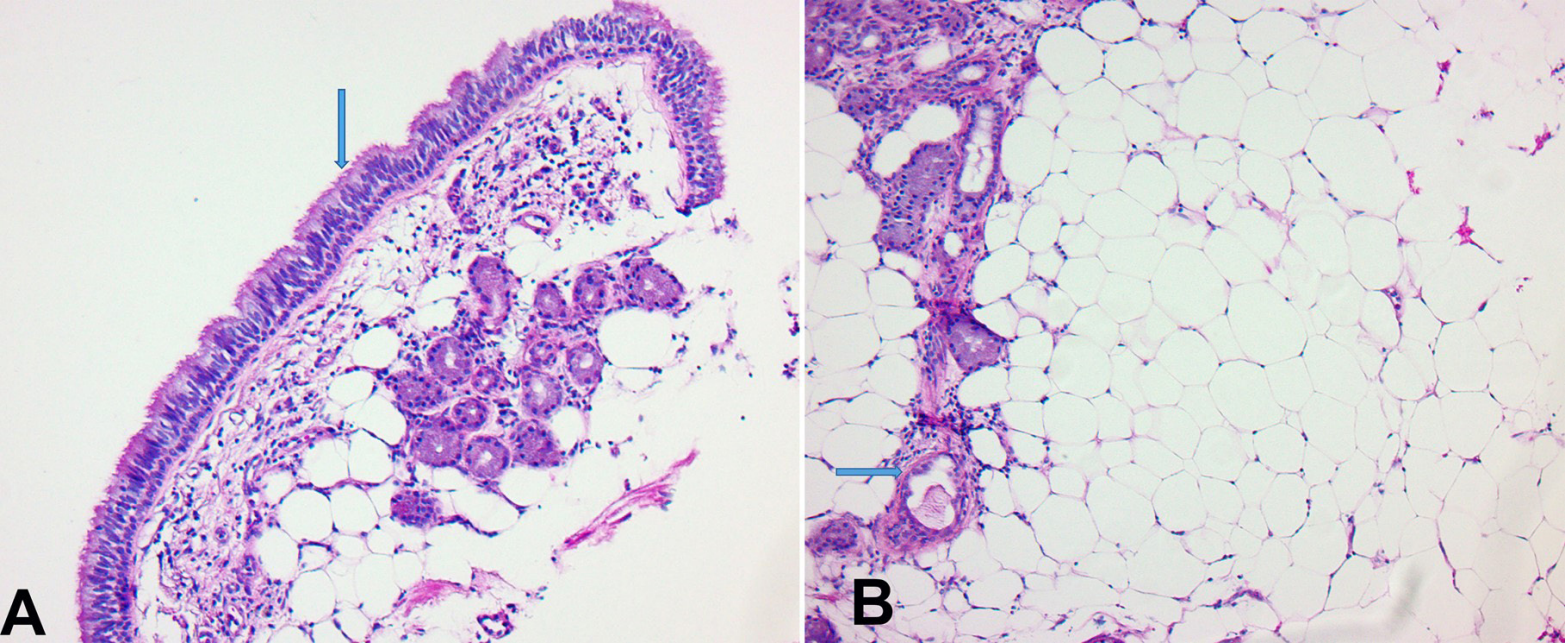
Pathology Report (A): H&E, 10X; Ciliated bronchial epithelium (arrow) with endobronchial glands and interspersed adipocytes. (B): H&E, 20X; Mature adipocytes, endobronchial glands and endobronchial vessels (arrow).

He underwent debulking using a cryotherapy probe followed by hemostasis control with an argon plasma coagulation probe; about 50% was resected. Histopathologic examination revealed ciliated bronchial epithelial lining underneath endobronchial glands and mature adipose tissue with no cartilaginous structure (Figure 2). Thus, the final diagnosis of endobronchial lipoma was rendered. On follow-up, no pulmonary nodules were noted.

## DISCUSSION

Endobronchial lipomas are rare. They have an incidence of 0.1 to 0.5% of all lung tumors.^1^^,^^33^ Though benign, it can cause bronchial obstruction and subsequent complications, such as early-onset dyspnea and wheezing. Unfortunately, their ultimate diagnosis can be easily overlooked, as providers may initially investigate the diagnosis of other more common obstructive pathologies (i.e., COPD, asthma). They usually present insidiously, most commonly in older men. Further symptoms can include dyspnea, cough, fever, chest pain, hemoptysis, and pneumonia.^3^ Management options can vary among cases of endobronchial lipomas, the most common and effective treatment being bronchoscopic resection. Among cases analyzed since 2010, according to one review, seventy three percent of endobronchial lipomas were resected bronchoscopically.^1^ Methods of bronchoscopic resection include cryotherapy, laser, electrosurgery, and mechanical debulking.^1^^,^^3^ According to case reports by Huisman et al.,^34^ electrocautery can also be used as an effective treatment. 

Upon review of 10 cases in this series,^3^^,^^6^^,^^7^^,^^9^^-^11^,^^25^^,^^26^^,^^28^^,^^30^ regardless of lipoma location, most of the diagnoses of endobronchial lipoma were secondary to presenting symptoms such as non-specific throat pain, shortness of breath upon exertion, and/or cough. These symptoms showed gradual resolution when the lipoma was resected. While an incidental diagnosis of endobronchial lipomas has been made, this is rather rare; diagnosis typically only occurs after the patient presents with relevant respiratory symptoms.

Similar to the cases seen in the literature review, our patient also presented with initial symptoms of labored breathing and chest pain. Unique to our case is the specific location of the mass. While the majority of cases presented in the right main or right lower lobe bronchi (Table 2), ours was located in the right middle lobe bronchus.

Our patient underwent debulking and cauterization, similarly following the trend of the other reported bronchoscopic mass resections seen in the case review. More invasive procedures, like lobectomies, were reserved for cases in which there was irreversible parenchymal damage, suspicion of diagnosis, or if bronchoscopic resection was not possible. Even though endobronchial lipoma is rare, it can mimic malignancy and lead to significant complications such as progressive dyspnea and subsequent lung infections related to endobronchial obstruction. There is a significant need to investigate endobronchial lesions as endobronchial lipoma should remain in the differential diagnosis.
